# Which potential harms and benefits of using ginger in the management of nausea and vomiting of pregnancy should be addressed? a consensual study among pregnant women and gynecologists

**DOI:** 10.1186/s12906-017-1717-0

**Published:** 2017-04-08

**Authors:** Ramzi Shawahna, Assim Taha

**Affiliations:** 1 Department of Physiology, Pharmacology and Toxicology, Faculty of Medicine and Health Sciences, An-Najah National University, New Campus, Building: 19, Office: 1340, P.O. Box 7, Nablus, Palestine; 2 An-Najah BioSciences Unit, Centre for Poisons Control, Chemical and Biological Analyses, An-Najah National University, Nablus, Palestine; 3 Department of Medicine, Faculty of Medicine and Health Sciences, An-Najah National University, Nablus, Palestine

**Keywords:** Ginger, Nausea and vomiting of pregnancy, Gynecologists, Pregnant women

## Abstract

**Background:**

Nausea and vomiting of pregnancy (NVP) affect approximately 80–90% of the pregnant women. Ginger (*Zingiber officinale* Roscoe) is the most widely used herbal therapy in the management of NVP. Like conventional therapies, herbal therapies have potential harms and benefits that patients need to be informed about in order to develop their therapy preferences. The aim of this study was to achieve consensus among women who suffered NVP and physicians often consulted by pregnant women on a core list of potential harms and benefits of using ginger to manage NVP to be addressed during clinical consultations.

**Methods:**

In this study, the Delphi technique was used to achieve consensus on a core list of important harms and benefits of using ginger in the management of NVP to be addressed during the clinical consultation. A Delphi process was followed in two panels in parallel sessions. One panel was composed of 50 gynecologists and other physicians who are often consulted by pregnant women suffering NVP and the other panel was composed of 50 women who suffered NVP.

**Results:**

Consensus was achieved on 21 (75%) of the 28 potential harms presented to the panelists. Panelists agreed that potential harms of the anticoagulant effects of ginger, risk with other co-morbidities, and risk of potential allergic reactions are important to address during the clinical consultation. Of the 14 potential benefits presented to the panelists in both panels, consensus was achieved on 13 (92.9%). Partial consensus on 7 potential harms and 1 potential benefit was achieved in both panels.

**Conclusions:**

Addressing important potential harms and benefits of using ginger for the management of NVP during the clinical consultations is important in promoting congruence and reducing patient dissatisfaction in clinical practice. Consensus was achieved on a core list of important harms and benefits of using ginger for the management of NVP to be addressed during the clinical consultations by a panel of women and a panel of physicians. Further studies are still needed to investigate what is being addressed during clinical consultations.

**Electronic supplementary material:**

The online version of this article (doi:10.1186/s12906-017-1717-0) contains supplementary material, which is available to authorized users.

## Background

Nausea and vomiting of pregnancy (NVP) rank high among the most common complaints during the early weeks of pregnancy [1]. In clinical practice, both patients and physicians are reluctant to use medications in pregnancy, especially in the first trimesters due to the possibility of harming the unborn fetus [2]. However, in many cases, NVP requires treatment, thus, leaving the pregnant and physician in a dilemma whether to use conventional medications or leave the condition untreated [1, 2]. Unfortunately, many pregnant women opt not to use conventional medications and thus are left helpless against the heavy burden of NVP. NVP affect approximately 80–90% of the pregnant women [3]. Typically, symptoms appear at 4–9 weeks of gestation, reaching a peak at 7–12 weeks, and often subside by week 16 [3]. However, in about 1 in 3 pregnant women, symptoms persist beyond 20 weeks or even throughout of the pregnancy [1, 2]. Many pregnant women might present a severer and more persistent form of vomiting known as *hyperemesis gravidarum* which can lead to dehydration, electrolyte disturbances, damage the liver, damage of the developing fetus, and in extreme cases, the death of the mother and her fetus. This condition occurs in nearly 2% of pregnancies [1, 2].

Treatment of NVP using conventional medications can be complicated because of the significant physiological changes occurring during the pregnancy such as those in the gastro-intestinal motility, plasma volume, and glomerular filtration [4]. Such changes would certainly affect the different pharmacokinetics of medications including absorption, distribution, metabolism, and excretion. Many medications are able to cross the placenta and reach the fetus. Therefore, not all medications are effective and safe in pregnancy. Herbal therapies have been traditionally regarded as alternatives to conventional medications. In recent years, there has been a growing interest in using herbal therapies to treat many conditions including NVP [2, 5]. Among these herbal therapies, ginger (*Zingiber officinale* Roscoe) is the most widely used herbal therapy in the management of NVP [2, 5–9].

The safety of herbal therapies has long been taken as granted. This believe might have emerged as herbal therapies are often advertised as gentle, safe, and possessing unique properties not found in other conventional medication therapies [10]. Unfortunately, some healthcare professionals have perpetuated this myth when recommending these herbal therapies as “natural”, thus, mistakenly understood as safe or at least safer than conventional medications [5, 11]. Today, many patients believe that herbal therapies can never be harmful. However, these claims are not true and lack scientific basis. Herbal therapies contain a wide range of chemicals that can be similar to the active ingredients in many conventional medication therapies. In this case, these chemicals act by the same pharmacological mechanism of action in the body and possess similar potential to cause adverse effects. Like conventional medication therapies, herbal therapies have their intended indications, contraindications, precautions and adverse effects. Ginger is no exception, and therefore, should be recommended for the right person, at the right time, in the right dose, at the right frequency, and by the right route of administration [11].

Ginger has been extensively used in the management of NVP. Scientific evidence on the effectives of ginger in managing NVP is still inconclusive in view of the conflicting reports regarding the evidence of its effectiveness [1, 6]. Moreover, prior studies showed that ginger was associated with many health related issues like decreasing platelet aggregation, increasing stomach acid production, herb-herb and herb-medication interactions [1, 12, 13]. Therefore, gynecologists and other physicians who are frequently consulted by pregnant women with NVP should discuss the potential harms and benefits of using ginger in case they opted for using ginger to manage NVP. Currently, the literature does not narrate intensively which potential harms and benefits of using ginger in the treatment of NVP should be addressed from the viewpoint of the women affected, gynecologists and other physicians who are frequently consulted by pregnant women suffering from NVP. The current study is proposed to fill this gap in the literature.

When opting for a treatment, in general, the potential benefits in terms of local control should be balanced against the potential harms, taking into account the available alternatives and patient preferences. In today’s clinical practice, patients need to be informed of the most relevant potential harms and benefits of the treatment options in order to develop their preferences [14]. Informing patients would probably prevent overestimation of the impact of treatment on cure [15]. It has also been suggested that well-informed patients experience better health-related quality of life and might cope better with the adverse effects of the treatment [16, 17]. In order to assess congruence with daily clinical practice, consensus was sought among pregnant women, gynecologists and other physicians who are frequently consulted by pregnant women for their NVP on which potential harms and benefits of using ginger for the management of NVP should be addressed during the consultations. In general, there are no recommendations on which potential harms and benefits of using ginger in the management of NVP to communicate to patients. Therefore, the aim of this study was to achieve consensus among women who suffered NVP, gynecologists and other physicians who are frequently consulted by pregnant women for their NVP on a core list of potential harms and benefits of using ginger to manage NVP that should be addressed during clinical consultations on which a decision to use ginger is taken.

## Methods

### Potential harms and benefits of using ginger in NVP

Prior to the iterative Delphi rounds, we interviewed 8 key contact gynecologists who frequently recommend pregnant women with NVP to use ginger and 8 women with more than 5 prior pregnancies who were recommended to use ginger to reduce the symptoms of their NVP. The gynecologists were asked to list the potential harms and benefits of using ginger in the treatment of NVP that should be addressed during the clinical consultation in which they advise their patients to use ginger. The women were asked to list the potential harms and benefits of using ginger in the management of NVP that they would like their physicians to address during the clinical consultation. The aim of these interviews were to generate an extensive list of potential harms and benefits of using ginger in the management of NVP. The potential harms and benefits provided by the interviewed gynecologists and women were noted. We then conducted an extensive literature review to identify potential harms and benefits of using ginger in pregnant women [1–3, 6–8, 11, 12, 18–35]. All potential harms and benefits provided by the gynecologists and women as well as those found in the literature were summarized, formulated into statements, and included into a questionnaire. Potential harms and benefits were ordered by the effect of ginger on the health of the pregnant woman or her fetus. Harms and benefits related to costs, convenience or inconvenience were excluded from the list. The questionnaire was piloted with five students of medicine and five lay persons for readability and comprehensibility. Some statements were edited to promote understanding.

### The Delphi technique

In this observational study, the Delphi technique was used to achieve consensus on the potential harms and benefits of using ginger in the management of NVP that should or should not be addressed in the clinical consultation. Since its inception, the Delphi technique has emerged as one of the most commonly used formal consensus techniques in healthcare on subjects with no or limited consensus [36, 37]. The Delphi technique is a combination of qualitative and quantitative approaches in which a multiple-round questionnaire system is administered in iterative rounds over an extended period of time within a panel until consensus is achieved [38]. In other words, items on which consensus was not achieved are often included in a revised questionnaire and presented to the panelists for further subsequent rounds [37]. Statistical summaries and comments made by one panelist are shared with other panelists in an attempt to reduce the number of rounds required to achieve consensus. Panelists are often requested to reconsider their voting in view of the votes and comments of other panelists [36]. In this study, we anticipated differences in views and opinions of physicians and women, therefore, we aimed to achieve consensus in two panels [14, 39]. One panel was composed of gynecologists and other physicians who are frequently consulted by pregnant women with NVP and another panel composed of women who suffered NVP. The Delphi technique was performed in the two panels separately and in paralleled sessions. The study was conducted between November 2016 and February 2017. As in previous Delphi consensus studies [14, 40, 41], we decided to achieve consensus in two consecutive iterative Delphi rounds.

### Panel of physicians

We used a purposive sampling method to recruit and compose a panel of gynecologists and other physicians who are frequently consulted by pregnant women with NVP. Personal contacts in the field were used to identify potential participants. As pregnant women suffering NVP often consult gynecologists, the panel included a large percentage of gynecologists. Selection of the panelists is one of the most critical steps in the Delphi technique as panelists should be rich with information and experience [42]. In this study, the inclusion of panelists was based on their qualifications and experience in the field of treating pregnant women with NVP. Potential participants were approached in person and invited to take part in the study. The design and objectives of the study were explained to potential participants and their consent was obtained before they took part. The inclusion criteria was as follows: 1) possession of a basic or advance degree in medicine, 2) licensed to practice medicine in Palestine, 3) at least 5 years of practicing experience in a healthcare setting attended by pregnant women with NVP, and as prior knowledge of the subject being investigated is a prerequisite for panelists in a Delphi technique, 4) knowledge of the use of ginger in managing NVP. In this study a total of 50 physicians were recruited to the panel. Panelists agreed to participate without any incentives.

### Panel of women

We used a snowball sampling technique to recruit women who were advised to use ginger for the management of their NVP. Personal contacts in the field helped identifying and recruiting potential participants who were approached in person and invited to take part in the study. The design and objectives of the study were explained to potential participants. Verbal consents were taken from all women before participation. The inclusion criteria was as follows: 1) multiparous, 2) suffered NVP, 3) was recommended to use ginger for her NVP, and 4) willingness to participate in the study. In this study, a total of 50 women were recruited to the panel. Again, the panelists agreed to participate without any financial incentives.

#### The first Delphi iterative round

In this round, the questionnaire was hand-delivered to 50 physicians and 50 women. The questionnaire contained three parts. In the first part, panelists were asked to provide their sociodemographic and practice characteristics. Physicians were asked to provide their gender, age, qualification, specialty, number of years in practice, place of work, if they recommend herbal therapies for pregnant women suffering NVP, and if they address potential harms and benefits of herbal therapies that pregnant women could be using during the clinical consultations. On the other hand, women were asked to provide their age, the number of pregnancies they had, any history of miscarriage, their educational level, their employment status, if they have been recommended by their physicians to use herbal therapies for their NVP, and if they like to have enough discussion with their physicians on the potential harms and benefits of using herbal therapies during the clinical consultations. The second part of the questionnaire contained 28 statements on potential harms from using ginger to manage NVP. The third part contained 14 potential benefits of using ginger in pregnant women. Both physicians and women were asked to indicate the level of their agreement and disagreement of the importance of addressing or not addressing the potential harm or benefit on a Likert scale of 9-points. Voting 1–3 indicated disagreement of the panelist on the importance of addressing the potential harm or benefit, i.e. it is not important to address the potential harm or benefit during the clinical consultation. Voting 7–9 indicated agreement of the panelist with on the importance of addressing the potential harm or benefit, i.e. it is important to address the potential harm or benefit during the clinical consultation. Voting 4–6 indicated that the panelist partially agrees on the importance of addressing the potential harm or benefit, i.e. the opinion of the panelist is inconclusive if it is important to address the potential harm or benefit during the clinical consultation. Panelists were encouraged to include written comments to justify or qualify their votes.

#### Analysis of the votes

Data obtained in the first Delphi iterative round were analyzed using descriptive statistics. Data were entered into an Excel Sheet (Microsoft Excel 2007). The first quartile (Q1), median (Q2), third quartile (Q3), and the interquartile range (IQR) of the votes were computed for each statement. Physicians and women were considered two different panels. Consensus was defined as in previous studies on issues in healthcare [40, 43]. When the median votes was between 7 and 9 and the IQR was between 0 and 2, consensus was said to have been achieved and the potential harm or benefit was included in the list of potential harms and benefits to be addressed during the clinical consultation. When the median vote was between 1 and 3 and the IQR was between 0 and 2, consensus was said to have been achieved and the potential harm or benefit was excluded from the list of potential harms and benefits to be addressed during the clinical consultation. When the median vote was between 4 and 6 or the IQR was larger than 2, the potential harm or benefit was considered equivocal. Consensus was based on the votes of at least 75% of the panelists in each panel.

#### The second Delphi iterative round

Potential harms and benefits that were considered equivocal in the first Delphi iterative round were included in a revised questionnaire. The questionnaire was hand-delivered to the panelists in a second Delphi iterative round. The panelists were provided with the followings: 1) the median vote on each equivocal statement along with the IQR, 2) reminder of their own vote, and 3) summary of the comments made by other panelists on the statement to justify or qualify their votes. The panelists were requested to re-consider their votes in view of the votes and comments of other panel members. It is believed that inclusion of such statistics and summaries reduce the number of rounds required to achieve consensus on issues in healthcare [36]. Votes obtained in the second Delphi iterative round were analyzed as in the first Delphi iterative round. Based on the voting and comments made by the panelists in both panels, it was decided that consensus would not be achieved in a further iterative round. Therefore, we decided not to conduct further rounds.

### Ethics

The protocol of this study received approval from the Institutional Review Board (IRB) committee of An-Najah National University (Protocol # 02-NOV-2016). All participants gave verbal consents before participation in this study. Views and opinions of all participants weighed equally in the analysis. Data were made anonymous before analysis. During the Delphi iterative rounds, each panelist remained anonymous to the rest of the panelists.

## Results

In the first Delphi iterative round, questionnaires were returned by the 50 physicians and 50 women, giving a response rate of 100%. However, in the second Delphi iterative round, questionnaires were returned by 43 (86%) physicians and 45 (90%) women.

### Characteristics of the panelists

The panel of physicians included practitioners of both genders, from different specialties, different geographical locations, belonged to different age groups, and with variable number of years in practice. The vast majority of the panelists were gynecologists. The sociodemographic and practice details of the physicians are presented in Table 1.

**Table 1:** Sociodemographic and practice characteristics of the physicians who participated in this study (*n = 50*)

Characteristic	Number of participants	Percent
Gender		
Male	29	58
Female	21	42
Age (years)		
< 40	27	54
≥ 40	23	46
Qualifications/specialty		
MD/PhD	4	8
MD/Obstetrics & Gynecology	39	78
MD/Public health	7	14
Employer		
Private clinic	13	26
Hospital	30	60
Other healthcare organization (WHO, University, …etc)	7	14
Year of graduation/obtaining licensure		
1965–1979	3	6
1980–1994	13	26
1995–2009	21	42
2010-Present	13	26
Experience (years)		
≤ 5	15	30
6–10	13	26
11–20	11	22
≥ 21	11	22

*MD* Doctor of Medicine; *PhD* Doctor of Philosophy; *WHO* World Health Organization

The panel of women included women of different age groups, had a variable number of pregnancies, had history of miscarriage, different educational levels and employment status. The detailed characteristics of the women who took part in this study are shown in Table 2.

**Table 2:** Sociodemographic characteristics of the women who participated in this study (*n = 50*)

Characteristic	Number of participants	Percent
Age (years)		
< 30	14	28
≥ 30	36	72
Number of pregnancies		
1–4	36	72
> 4	14	28
History of miscarriage		
Yes	16	32
No	34	68
Educational level		
High school	16	32
University	34	68
Employment status		
Not employed (housewife)	16	32
Healthcare establishment	13	26
Educational establishment	17	34
Governmental organization/other	4	8

### Use of ginger for NVP

When the physicians were asked if they recommend herbal therapy for pregnant women with NVP, 62% of them responded by quite often and the rest of 38% responded by sometimes. When the women were asked if they have been recommended by their physicians to use herbal therapies for their NVP, 70% responded by quite often and 30% responded by sometimes.

When the physicians were asked if they address potential harms and benefits of herbal therapies that pregnant women could be using during the clinical consultation, 66% responded by quite often and 34% replied by sometimes. When the women were asked if they like to have enough discussion with their physicians on the potential harms and benefits of using herbal therapies, 76% responded by always and 24% responded by sometimes.

### Important potential harms and benefits to be addressed during the clinical consultation

Table 3 shows the important potential harms to be addressed during the clinical consultation on which consensus was achieved by the panelists.

**Table 3:** Important potential harms of using ginger for the management of NVP to be addressed during the clinical consultation

		Physicians				Women			
		Iterative round 1		Iterative round 2		Iterative round 1		Iterative round 2	
Item #	Potential harms	M	IQR	M	IQR	M	IQR	M	IQR
	Anticoagulant effects								
1	Pregnant women at risk of bleeding should be warned of the anticoagulant effects of ginger	6.5	2.0	7.0	1.8	8.0	1.8	NA	NA
2	Pregnant women with history of clotting/bleeding disorders should be warned of the anticoagulant effects of ginger	7.0	2.5	7.0	2.0	8.0	2.0	NA	NA
3	Pregnant women with history of vaginal bleeding should be warned of the anticoagulant effects of ginger	7.0	1.8	NA	NA	8.0	2.0	NA	NA
4	Pregnant women close to labor should be warned of the anticoagulant effects of ginger	6.0	4.0	7.0	1.0	7.0	2.0	NA	NA
5	Pregnant women taking anticoagulants should be warned of the anticoagulant effects of ginger	7.0	2.8	7.5	2.0	7.0	1.8	NA	NA
6	Pregnant women taking non-steroidal anti-inflammatory drugs (NSAIDs) should be warned of the anticoagulant effects of ginger	5.0	4.0	7.0	2.0	7.0	3.0	7.0	0.5
	Risk of abortion								
7	Pregnant women should be warned that ginger may be associated with spontaneous abortion in some pregnancies	5.0	4.0	7.0	2.0	8.0	2.0	NA	NA
8	Pregnant women with a history of miscarriage should be warned of increasing risk of abortion associated with taking ginger	5.0	4.0	7.0	2.0	7.0	2.0	NA	NA
9	Pregnant women should be warned that ginger may be associated with impairment of fetal development	3.0	4.8	7.0	2.0	7.0	3.0	7.0	0.5
	Risk of other co-morbidities								
10	Pregnant women should be warned that ginger may be associated with cardiac arrhythmias	5.0	5.0	7.0	2.0	7.0	2.8	7.0	0.5
11	Pregnant women should be warned that ginger may stimulate irritable bowel syndrome	6.0	3.0	7.0	1.5	7.0	2.5	7.0	1.0
12	Pregnant women should be warned that ginger may stimulate duodenal ulcer	6.0	3.0	7.0	2.0	7.0	3.0	7.0	1.0
13	Pregnant women should be warned that ginger may stimulate the secretion of bile and should be avoided in people with a history of gallstones	5.0	3.0	7.0	2.0	7.0	2.0	NA	NA
14	Pregnant women should be warned that ginger may induce heartburn	7.0	2.0	NA	NA	7.0	2.0	NA	NA
	Ginger lowers blood pressure								
15	Pregnant women with a history of hypotension should be warned that ginger might reduce their blood pressure	6.0	4.0	7.0	2.0	7.0	2.0	7.0	0.5
16	Pregnant women with a history of dizziness should be warned that ginger might reduce their blood pressure and worsen their dizziness	5.0	4.0	7.0	1.0	7.0	2.0	NA	NA
17	Pregnant women taking anti-hypertensive medications should be warned that ginger might further reduce their blood pressure	5.5	3.8	7.0	0.8	7.0	2.0	NA	NA
	Ginger lowers blood sugar								
18	Pregnant women with a history of hypoglycemia should be warned that ginger might further reduce their blood sugar	6.5	2.8	7.0	2.0	7.0	2.0	NA	NA
19	Diabetic pregnant women whose diabetes is controlled by medications or insulin should be warned that ginger might reduce their blood sugar	5.0	4.0	7.0	1.0	7.0	2.0	NA	NA
	Other adverse effects								
20	Pregnant women should be warned that ginger may induce some allergic reactions	6.0	3.0	7.0	2.0	6.0	2.0	7.0	1.0
21	Pregnant women should be warned that ginger may cause dehydration	5.0	3.8	7.0	2.0	6.0	2.8	7.0	2.0

*IQR* interquartile range; *M* median; *NA* not applicable

**Fig. 1:**
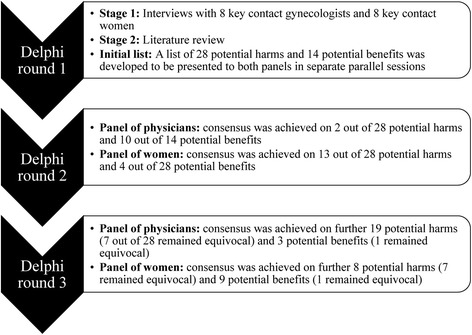
Details of the Delphi iteration process

By the end of the second iterative round, consensus was achieved on 21 (75%) of the 28 potential harms presented to the panels. The details of the Delphi iteration are shown in Fig. 1.

Panelists agreed that potential harms of the anticoagulant effects of ginger, risk with other co-morbidities, and risk of potential allergic reactions are important to address during the clinical consultation.

Of the 14 potential benefits presented to the panelists in both panels, consensus was achieved on 13 (92.9%). These potential benefits are shown in Table 4.

**Table 4:** Important potential benefits of using ginger for the management of NVP to be addressed during the clinical consultation

		Physicians				Women			
		Iterative round 1		Iterative round 2		Iterative round 1		Iterative round 2	
Item #	Potential benefits	M	IQR	M	IQR	M	IQR	M	IQR
1	Pregnant women could be informed that ginger can be beneficial for pregnancy-associated nausea and vomiting	8.0	1.75	NA	NA	7.5	1.8	NA	NA
2	Pregnant women could be informed that ginger can be beneficial for nausea and vomiting in motion sickness	8.0	1.75	NA	NA	8.0	1.0	NA	NA
3	Pregnant women could be informed that ginger may be beneficial for relieve of cough	7.0	1.75	NA	NA	7.0	2.8	7.0	0.5
4	Pregnant women could be informed that ginger may be beneficial for relieve of flu	7.0	3	7.0	2	7.0	2.8	7.0	0.5
5	Pregnant women could be informed that ginger may be beneficial for relieve of chronic pulmonary diseases	7.0	3	7.0	2	7.0	2.0	NA	NA
6	Pregnant women could be informed that ginger may enhance their natural milk production	7.0	2	NA	NA	7.0	3.0	7.0	1.0
7	Pregnant women could be informed that ginger may be beneficial for reducing chronic joint pain	7.0	2	NA	NA	7.0	3.0	7.0	0.5
8	Pregnant women could be informed that ginger may be beneficial for their skin health	7.0	2	NA	NA	7.0	3.0	7.0	1.0
9	Pregnant women could be informed that ginger may be beneficial in decreasing appetite in case of eating disorders	7.0	2	NA	NA	7.0	2.8	7.0	2.0
10	Pregnant women could be informed that ginger may promote weight loss	7.0	1.75	NA	NA	7.0	2.8	7.0	2.0
11	Pregnant women could be informed that ginger may decrease cholesterol levels	7.0	2.75	7.0	0.5	7.0	1.0	NA	NA
12	Pregnant women could be informed that ginger may help enhance diuresis	7.0	2	NA	NA	7.0	2.5	7.0	1.0
13	Pregnant women could be informed that ginger may be beneficial in functional dyspepsia	7.0	2	NA	NA	7.0	3.0	7.0	0.3

*IQR* interquartile range; *M* median; *NA* not applicable

Partial consensus on 7 potential harms and 1 potential benefit was achieved by both panels. These potential harms and benefits are presented in Table 5. The choice to address these issues or not was left to the clinician depending on the individual clinical situation’s need.

**Table 5:** Potential harms and benefits to be addressed or not during a clinical consultation depending on the individual clinical situation’s need

		Physicians				Women			
		Iterative round 1		Iterative round 2		Iterative round 1		Iterative round 2	
Item #	Potential harm	M	IQR	M	IQR	M	IQR	M	IQR
	Risk of other co-morbidities								
1	Pregnant women should be warned that ginger may induce diarrhea	4.0	4.8	4.0	5.0	6.5	2.0	6.0	1.0
2	Pregnant women should be warned that ginger may cause mild headache	5.0	4.0	6.0	2.0	6.0	2.0	6.0	2.0
	Other adverse effects								
3	Pregnant women should be warned that ginger may induce fever	5.5	4.0	5.5	2.5	6.0	2.8	6.0	3.0
4	Pregnant women should be warned that ginger may induce sweating	6.0	2.0	6.0	1.0	6.0	2.0	6.0	0.5
5	Pregnant women should be warned that ginger may induce thirst	7.0	3.0	7.0	3.0	6.0	3.0	6.0	0.8
6	Pregnant women should be warned that ginger may induce mild skin itching	5.0	4.0	5.0	0.0	5.0	3.0	5.0	0.5
7	Pregnant women should be warned that ginger may induce belching	6.0	3.0	6.0	2.0	6.0	3.0	6.0	1.0
	Potential benefits								
1	Pregnant women could be informed that ginger may induce somnolence	5.0	3.0	5.0	0.5	5.0	2.0	5.0	2.0

*IQR* interquartile range; *M*: median

## Discussion

In this study we sought consensus on a list of important potential harms and benefits of using ginger for the management of NVP that should be addressed during the clinical consultation in the Palestinian clinical practice by a panel of physicians and a panel of women. To the best of our knowledge, this is the first attempt to achieve consensus on such list of potential harms and benefits of ginger in NVP using formal consensus techniques.

In this study, we used a purpose sampling technique to recruit the panel of physicians and a snowball sampling technique to recruit the panel of women. In conservative views, these sampling techniques has long been considered biased [44, 45]. However, using other randomized sampling techniques was not possible in this study as these techniques are not suitable for the type of this study. Using these sampling techniques allowed the inclusion of panel members who had prior knowledge of the subject being investigated. In this study, we recruited physicians the majority of which were gynecologists and the rest were physicians who are frequently consulted by pregnant women suffering from NVP. Currently, there is no consensus on the ideal number of panelists in a Delphi panel. Previous studies used panels ranging in size from 10 to 1000 [44]. The number of panelists in this study was larger or similar to those previously used in Delphi consensus studies on issues in healthcare [40, 41, 46]. The advantages of the Delphi technique includes maintaining anonymity of the panelists, possibility of including panelists from different geographic locations, reduces costs and efforts to bring the panelists together compared to focus groups, and ensures immunity against individual domination of the decision compared to nominal or focused groups [44, 47].

The aim of this study was to achieve consensus on a list of the important potential harms and benefits of using ginger for the management of NVP that should be addressed during the clinical consultation. This list would be used by clinicians as a guidance on what potential harms and benefits to address during the clinical consultation. Guidelines on what clinicians should address during the clinical consultation when ginger is advised for NVP do not exist. We, therefore, believe that such lists developed through consensual methods might be beneficial in changing the behavior of physicians during the clinical consultation [14, 40, 43, 46, 48–50].

Prior studies reported high usage of herbal therapies in the Palestinian population including women who were pregnant [51–53]. In this study, 70% of the women reported being quite often recommended to use herbal therapies for their NVP. Similarly, 62% of the physicians admitted recommending quite often herbal therapies for pregnant women suffering NVP. It was reported that about 50% of the users of herbal therapies would not inform their physicians of such use [54]. Similarly, another study reported that physicians seldom ask if the patient was using herbal therapies [55]. Therefore, many patients end up using herbal and conventional therapies concurrently [12]. This has been attributed to poor communication and the insufficient time allocated to the clinical consultation [56]. The panel of women included women who had previous pregnancies, suffered NVP, and used ginger to manage their NVP. Women included in the panel were expected to provide the concerns that pregnant women suffering NVP would like their physicians to address during the clinical consultation. Interestingly, 76% of the women wanted their physicians to address the potential harms and benefits of the herbal therapies during the clinical consultation.

High response rates in both Delphi iterative rounds from physicians and women is a major strength that adds to the validity of this study. Previous studies used panels that greatly varied in size ranging from 10 to more than 1000 participants [44, 57]. Studies using the Delphi technique to achieve consensus on issues in healthcare used panels of 50 participants or less [36, 46, 50]. In this study, both panels were composed of 50 members. The panel size used in this study was either comparable to or more than those used in previous studies [36, 40, 43, 46, 50]. The panel of physicians included participants of both genders, from different geographical locations, clinical practice settings, age groups, and experience periods. The panel of women also included participants from different geographical locations, age groups, number of pregnancies, history of miscarriage, employment, and educational levels. This diversity adds to the validity and suitability of addressing the potential harms and benefits that the participants agreed upon in this study. It has been argued that in absence of gold standards, consensual methods provide means of reducing bias, promoting transparency, and validity of judgmental methods when developing certain criteria [58]. Therefore, we believe that addressing these potential harms and benefits of using ginger for NVP during a clinical consultation approached using formal consensus method might be more appealing to clinical practitioners advising pregnant women to use ginger for their NVP.

Interestingly in this study, consensus was achieved on six potential harms associated with the potential anticoagulant effects of ginger that should be addressed during the clinical consultation by both women and physicians (Table 3). These findings are not surprising, as previous studies showed that patients wanted to hear more from their healthcare providers on the medications they are taking [59]. The American Society of Anesthesiologists has advised that patients should discontinue all herbal therapies 2 to 3 weeks before an elective surgical procedure to avoid any potential intraoperative adverse events [60]. Recently, Marx et al. systematically reviewed eight clinical trials and two observational studies on the anticoagulant effects of ginger [26]. Considering the risks of bias, methodological variation, timeframe studied, dose of ginger used, and characteristics of the participants, Marx et al. concluded that the evidence that ginger affects platelet aggregation and coagulation is still equivocal and further studies are needed to illustrate a definite conclusion. However, a previous study showed that gingerols, which are compounds found in ginger, and the related compounds were able to inhibit arachidonic acid-induced human platelet serotonin release and aggregation in vitro [25]. The potency of these compounds were comparable to aspirin. Another study showed that 8-paradol, which is a component of ginger, was a relatively potent COX-1 inhibitor and antiplatelet aggregation agent compared to four other components of ginger with antiplatelet activities [27]. In spite of the fact that the anticoagulant effects of ginger are still inconclusive. Bleeding in the first trimester of pregnancy can have detrimental effects on the mother and her fetus. Hasan et al. reported association between heavy bleeding in the first trimester, especially when accompanied with pain, and higher risk of miscarriage in a study with 4539 women [61]. In this study, panelists were of the opinion that physician should address the potential anticoagulant effects of ginger with pregnant women who are at higher risk of bleeding to make a better informed decision whether to use ginger or not.

Both physicians and women agreed that the risks associated with abortion should also be addressed during the clinical consultation when pregnant women are advised to take ginger for their NVP (Table 3, items 7–9). Today, there is no conclusive evidence of the adverse effects of ginger on the developing fetus. Therefore, ginger and ginger containing products are labeled differently across the globe. In the United States, ginger is “generally regarded as safe”. However, in Germany, the German E Commission on herbal medicines (does not exist anymore) recommended that ginger to be avoided in pregnancy [11]. Moreover, the Finnish Food Safety Authority Evira recommended that ginger products, ginger tea, and food supplements containing ginger should bear a warning label as not recommended during pregnancy [18]. Previous studies showed that ginger might be associated with spontaneous abortion and impairment of fetal development [21–23, 30]. Portnoi et al. conducted a study in Canada in which the birth outcomes of 187 women who were exposed to ginger in their first trimester of pregnancy were prospectively compared to the birth outcomes of 187 women who were exposed to other nonteratogenic medications that were not antiemetics [29]. The comparison showed that there was no statistically significant difference in terms of live births, spontaneous abortions, therapeutic abortions, birth weight, and/or gestational age between both groups. More recently, Heitmann et al. reported on the safety of using ginger during pregnancy in terms of congenital malformations and selected pregnancy outcomes in a large cohort of 68,522 women in Norway [24]. The study showed that 1020 women which represented 1.5% of the study population used ginger during their pregnancies. The study concluded that there was no increased risk of stillbirth/perinatal death, preterm birth, low birth weight, or low Appearance, Pulse, Grimace, Activity, Respiration (Apgar) score for the women who were exposed and those who were not exposed to ginger. Taking a conservative approach, women should be warned of the still inconclusive association between exposure to ginger and risk on the fetus and continuity of the pregnancy.

Ginger could be associated with or could worsen symptoms of other co-morbidities [19, 20, 28, 31]. Ginger might be associated with reducing blood pressure and blood sugar. Ginger can cause dehydration and allergic reactions. In this study, both physicians and women agreed that such possibilities should be addressed during the clinical consultation. Pregnant women should be warned that ginger might precipitate cardiac arrhythmias, stimulate irritable bowel syndrome, duodenal ulcer, secretion of bile, and heartburn. Physicians should address these potential harms during the clinical consultation. For example, pregnant women at risk of cardiac arrhythmias or those taking antiarrhythmic medications might be advised not to take ginger and a suitable alternative might be recommended. Similar measures should be applied to avoid the potential harm of ginger in worsen other conditions.

The views of both physicians and women were divisive whether to address the potentials of ginger inducing diarrhea, mild headache, fever, sweating, thirst, mild skin itching, and belching. It is noteworthy to mention that in many studies the seriousness of the reported adverse effects depends on the subjective judgements of the research team taking into account the possibility of these events in normal pregnancies without any interventions [1]. Classifying the potential harms of ginger into major and minor goes beyond the scope of this study, however in general, researchers often classify harms as major when the consequence was serious or detrimental to the mother and/or fetus. When the harm was a merely discomfort and manageable it was considered minor.

Both physicians and women agreed that pregnant women suffering NVP might be informed of the potential benefits of ginger for NVP, nausea and vomiting in motion sickness, cough, flu, chronic pulmonary disease, milk production, joint pain, skin health, appetite in eating disorders, weight loss, hypercholesterolemia, diuresis, and dyspepsia. Physicians and women were divisive whether to address that ginger might induce somnolence.

When pregnant women need treatment, more care should be exerted when prescribing medications to this vulnerable group of patients. The risks should be weighed against the benefits of using a specific treatment considering the available alternatives and consequences of using or not using these treatments. The same measures should be applied when advising them to take herbal therapies.

## Conclusion

Addressing important potential harms and benefits of using ginger for the management of NVP during the clinical consultations is important in promoting congruence and reducing patient dissatisfaction in clinical practice. In this study, consensus was achieved on a list of important potential harms and benefits of using ginger for the management of NVP to be addressed during the clinical consultations by a panel of women and a panel of gynecologists and other physicians who are frequently consulted by pregnant women with NVP. This list might serve as a guidance for clinicians on what to address with their patients when recommending ginger for NVP. Some potential harms and benefits were divisive among women and physicians, either they should be addressed or not. The decision to whether to address them or not was left to the clinicians and on the needs of each clinical situation, i.e. to be evaluated on case-by-case basis. The use of such consensual core lists might promote congruence and reduce patient dissatisfaction in clinical practice. More randomized double blind controlled studies are needed to establish the efficacy and safety of ginger. Further studies are still needed to investigate what is being addressed during clinical consultations.

## Additional files

Additional file 1: Table S1. The questionnaire used for physicians. (DOCX 27 kb)

Additional file 2: Table S2. The questionnaire used for women. (DOCX 27 kb)

## Abbreviations

IRB: Institutional Review Board
NVP: Nausea and vomiting of pregnancy
