# Supplementary material for: Which potential harms and benefits of using ginger in the management of nausea and vomiting of pregnancy should be addressed? a consensual study among pregnant women and gynecologists
Source: BMC Complement Altern Med. 2017 Apr 8;17:204. doi: 10.1186/s12906-017-1717-0 (PMC5385053; doi:10.1186/s12906-017-1717-0)
Supplement: Supplementary file 1 — The questionnaire used for physicians. (DOCX 27 kb) [file 12906_2017_1717_MOESM1_ESM.docx]

1. Gender: □ Male □ Female
2. Age (years): ………...........................
3. Qualifications: ………………………………………
4. When did you graduate with the first degree? …………………………………………
5. Specialty: ……………………………………………….
6. Number of years in practice? ………………………………………………
7. Place of work? ……………………………………………………
8. Do you recommend herbal therapy for pregnant women with nausea and vomiting of pregnancy? □ Quite often □ Sometimes □ Never
9. Do you address potential harms and benefits of herbal therapies that pregnant women could be using during the clinical consultation? □ Quite often □ Sometimes □ Never

|  | **Dangers** |  |  |  |  |  |  |  |  |  |
| --- | --- | --- | --- | --- | --- | --- | --- | --- | --- | --- |
|  |  | **Degrees of disagreement** | | | **Degrees of neutrality** | | | **Degrees of agreement** | | |
|  | **Ginger has anticoagulant effects** | I strongly disagree | I moderately disagree | I disagree | I am neutral but tend to disagree | I am neutral | I am neutral but tend to agree | I agree | I moderately agree | I strongly agree |
| **1** | Pregnant women with history of clotting disorders should be warned not to take ginger | 1 | 2 | 3 | 4 | 5 | 6 | 7 | 8 | 9 |
|  |  | Comment (optional): | | | | | | | | |
| **2** | Pregnant women with history of vaginal bleeding should be warned not to take ginger | 1 | 2 | 3 | 4 | 5 | 6 | 7 | 8 | 9 |
|  |  | Comment (optional): | | | | | | | | |
| **3** | Pregnant women at risk of bleeding should be warned not to take ginger | 1 | 2 | 3 | 4 | 5 | 6 | 7 | 8 | 9 |
|  |  | Comment (optional): | | | | | | | | |
| **4** | Pregnant women close to labor should be warned not to take ginger | 1 | 2 | 3 | 4 | 5 | 6 | 7 | 8 | 9 |
|  |  | Comment (optional): | | | | | | | | |
| **5** | Pregnant women taking anticoagulants should be warned not to take ginger | 1 | 2 | 3 | 4 | 5 | 6 | 7 | 8 | 9 |
|  |  | Comment (optional): | | | | | | | | |
| **6** | Pregnant women taking non-steroidal anti-inflammatory drugs (NSAIDs) should be warned not to take ginger | 1 | 2 | 3 | 4 | 5 | 6 | 7 | 8 | 9 |
|  | **Risk of abortion** | Comment (optional): | | | | | | | | |
| **7** | Pregnant women should be warned that ginger may be associated with spontaneous abortion in some pregnancies | 1 | 2 | 3 | 4 | 5 | 6 | 7 | 8 | 9 |
|  |  | Comment (optional): | | | | | | | | |
| **8** | Pregnant women with history of miscarriage should be warned not to take ginger | 1 | 2 | 3 | 4 | 5 | 6 | 7 | 8 | 9 |
|  |  | Comment (optional): | | | | | | | | |
| **9** | Pregnant women should be warned that ginger may be associated with impairment of fetal development | 1 | 2 | 3 | 4 | 5 | 6 | 7 | 8 | 9 |
|  | **Risk of other co-morbidities** | Comment (optional): | | | | | | | | |
| **10** | Pregnant women should be warned that ginger may be associated with cardiac arrhythmias | 1 | 2 | 3 | 4 | 5 | 6 | 7 | 8 | 9 |
|  |  | Comment (optional): | | | | | | | | |
| **11** | Pregnant women should be warned that ginger may stimulate irritable bowel syndrome | 1 | 2 | 3 | 4 | 5 | 6 | 7 | 8 | 9 |
|  |  | Comment (optional): | | | | | | | | |
| **12** | Pregnant women should be warned that ginger may stimulate duodenal ulcer | 1 | 2 | 3 | 4 | 5 | 6 | 7 | 8 | 9 |
|  |  | Comment (optional): | | | | | | | | |
| **13** | Pregnant women should be warned that ginger may induce diarrhea | 1 | 2 | 3 | 4 | 5 | 6 | 7 | 8 | 9 |
|  |  | Comment (optional): | | | | | | | | |
| **14** | Pregnant women should be warned that ginger may stimulate the secretion of bile and should be avoided in people with a history of gallstones | 1 | 2 | 3 | 4 | 5 | 6 | 7 | 8 | 9 |
|  |  | Comment (optional): | | | | | | | | |
| **15** | Pregnant women should be warned that ginger may induce heartburn | 1 | 2 | 3 | 4 | 5 | 6 | 7 | 8 | 9 |
|  |  | Comment (optional): | | | | | | | | |
| **16** | Pregnant women should be warned that ginger may cause headache | 1 | 2 | 3 | 4 | 5 | 6 | 7 | 8 | 9 |
|  |  | Comment (optional): | | | | | | | | |
| **17** | Pregnant women should be warned that ginger may cause dehydration | 1 | 2 | 3 | 4 | 5 | 6 | 7 | 8 | 9 |
|  | **Ginger lowers blood pressure** | Comment (optional): | | | | | | | | |
| **18** | Pregnant women with a history of hypotension should be warned not to take ginger | 1 | 2 | 3 | 4 | 5 | 6 | 7 | 8 | 9 |
|  |  | Comment (optional): | | | | | | | | |
| **19** | Pregnant women with a history of dizziness should be warned not to take ginger | 1 | 2 | 3 | 4 | 5 | 6 | 7 | 8 | 9 |
|  |  | Comment (optional): | | | | | | | | |
| **20** | Pregnant women taking anti-hypertensive medications should be warned not to take ginger | 1 | 2 | 3 | 4 | 5 | 6 | 7 | 8 | 9 |
|  | **Ginger lowers blood sugar** | Comment (optional): | | | | | | | | |
| **21** | Pregnant women with a history of hypoglycemia should be warned not to take ginger | 1 | 2 | 3 | 4 | 5 | 6 | 7 | 8 | 9 |
|  |  | Comment (optional): | | | | | | | | |
| **22** | Diabetic pregnant women whose diabetes is controlled by medications or insulin should be warned not to take ginger | 1 | 2 | 3 | 4 | 5 | 6 | 7 | 8 | 9 |
|  | **Other adverse effects** | Comment (optional): | | | | | | | | |
| **23** | Pregnant women should be warned that ginger may induce fever | 1 | 2 | 3 | 4 | 5 | 6 | 7 | 8 | 9 |
|  |  | Comment (optional): | | | | | | | | |
| **24** | Pregnant women should be warned that ginger may induce sweating | 1 | 2 | 3 | 4 | 5 | 6 | 7 | 8 | 9 |
|  |  | Comment (optional): | | | | | | | | |
| **25** | Pregnant women should be warned that ginger may induce thirst | 1 | 2 | 3 | 4 | 5 | 6 | 7 | 8 | 9 |
|  |  | Comment (optional): | | | | | | | | |
| **26** | Pregnant women should be warned that ginger may induce skin itching | 1 | 2 | 3 | 4 | 5 | 6 | 7 | 8 | 9 |
|  |  | Comment (optional): | | | | | | | | |
| **27** | Pregnant women should be warned that ginger may induce some allergic reactions | 1 | 2 | 3 | 4 | 5 | 6 | 7 | 8 | 9 |
|  |  | Comment (optional): | | | | | | | | |
| **28** | Pregnant women should be warned that ginger may induce belching | 1 | 2 | 3 | 4 | 5 | 6 | 7 | 8 | 9 |
|  | **Benefits** | Comment (optional): | | | | | | | | |
| **1** | Pregnant women could be informed that ginger can be beneficial for nausea and vomiting in pregnancy | 1 | 2 | 3 | 4 | 5 | 6 | 7 | 8 | 9 |
|  |  | Comment (optional): | | | | | | | | |
| **2** | Pregnant women could be informed that ginger can be beneficial for nausea and vomiting in motion sickness | 1 | 2 | 3 | 4 | 5 | 6 | 7 | 8 | 9 |
|  |  | Comment (optional): | | | | | | | | |
| **3** | Pregnant women could be informed that ginger does not appear to increase the rates of major malformations | 1 | 2 | 3 | 4 | 5 | 6 | 7 | 8 | 9 |
|  |  | Comment (optional): | | | | | | | | |
| **4** | Pregnant women could be informed that ginger does not appear to increase the risks of congenital abnormalities | 1 | 2 | 3 | 4 | 5 | 6 | 7 | 8 | 9 |
|  |  | Comment (optional): | | | | | | | | |
| **5** | Pregnant women could be informed that ginger does not appear to increase the risks of fetal deaths | 1 | 2 | 3 | 4 | 5 | 6 | 7 | 8 | 9 |
|  |  | Comment (optional): | | | | | | | | |
| **6** | Pregnant women could be informed that ginger does not appear to increase the risks of low birth weights | 1 | 2 | 3 | 4 | 5 | 6 | 7 | 8 | 9 |
|  |  | Comment (optional): | | | | | | | | |
| **7** | Pregnant women could be informed that ginger does not appear to increase the risks of low Apgar scores | 1 | 2 | 3 | 4 | 5 | 6 | 7 | 8 | 9 |
|  |  | Comment (optional): | | | | | | | | |
| **8** | Pregnant women could be informed that ginger does not appear to increase the risks of low Apgar scores | 1 | 2 | 3 | 4 | 5 | 6 | 7 | 8 | 9 |
|  |  | Comment (optional): | | | | | | | | |
| **9** | Pregnant women could be informed that ginger may be beneficial for relieve of cough | 1 | 2 | 3 | 4 | 5 | 6 | 7 | 8 | 9 |
|  |  | Comment (optional): | | | | | | | | |
| **10** | Pregnant women could be informed that ginger may be beneficial for relieve of flu | 1 | 2 | 3 | 4 | 5 | 6 | 7 | 8 | 9 |
|  |  | Comment (optional): | | | | | | | | |
| **11** | Pregnant women could be informed that ginger may be beneficial for relieve of chronic pulmonary diseases | 1 | 2 | 3 | 4 | 5 | 6 | 7 | 8 | 9 |
|  |  | Comment (optional): | | | | | | | | |
| **12** | Pregnant women could be informed that ginger may enhance their natural milk production | 1 | 2 | 3 | 4 | 5 | 6 | 7 | 8 | 9 |
|  |  | Comment (optional): | | | | | | | | |
| **13** | Pregnant women could be informed that ginger may be beneficial for reducing chronic joint pain | 1 | 2 | 3 | 4 | 5 | 6 | 7 | 8 | 9 |
|  |  | Comment (optional): | | | | | | | | |
| **14** | Pregnant women could be informed that ginger may be beneficial for their skin health | 1 | 2 | 3 | 4 | 5 | 6 | 7 | 8 | 9 |
|  |  | Comment (optional): | | | | | | | | |
| **15** | Pregnant women could be informed that ginger may be beneficial in decreasing appetite in case of eating disorders | 1 | 2 | 3 | 4 | 5 | 6 | 7 | 8 | 9 |
|  |  | Comment (optional): | | | | | | | | |
| **16** | Pregnant women could be informed that ginger may promote weight loss | 1 | 2 | 3 | 4 | 5 | 6 | 7 | 8 | 9 |
|  |  | Comment (optional): | | | | | | | | |
| **17** | Pregnant women could be informed that ginger may decrease cholesterol levels | 1 | 2 | 3 | 4 | 5 | 6 | 7 | 8 | 9 |
|  |  | Comment (optional): | | | | | | | | |
| **18** | Pregnant women could be informed that ginger may help in induction of labor for women who has postdate pregnancies | 1 | 2 | 3 | 4 | 5 | 6 | 7 | 8 | 9 |
|  |  | Comment (optional): | | | | | | | | |
| **19** | Pregnant women could be informed that ginger may help enhance diuresis | 1 | 2 | 3 | 4 | 5 | 6 | 7 | 8 | 9 |
|  |  | Comment (optional): | | | | | | | | |
| **20** | Pregnant women could be informed that ginger may induce somnolence | 1 | 2 | 3 | 4 | 5 | 6 | 7 | 8 | 9 |
|  |  | Comment (optional): | | | | | | | | |
| **21** | Pregnant women could be informed that ginger does not increase cesarean section rates | 1 | 2 | 3 | 4 | 5 | 6 | 7 | 8 | 9 |
|  |  | Comment (optional): | | | | | | | | |
| **22** | Pregnant women could be informed that ginger does not increase stillbirth rates | 1 | 2 | 3 | 4 | 5 | 6 | 7 | 8 | 9 |
|  |  | Comment (optional): | | | | | | | | |
| **23** | Pregnant women could be informed that ginger does not increase neonatal death rates | 1 | 2 | 3 | 4 | 5 | 6 | 7 | 8 | 9 |
|  |  | Comment (optional): | | | | | | | | |
| **24** | Pregnant women could be informed that ginger does not increase preterm birth rates | 1 | 2 | 3 | 4 | 5 | 6 | 7 | 8 | 9 |
|  |  | Comment (optional): | | | | | | | | |
| **25** | Pregnant women could be informed that ginger may be beneficial in functional dyspepsia | 1 | 2 | 3 | 4 | 5 | 6 | 7 | 8 | 9 |
|  |  | Comment (optional): | | | | | | | | |

**Thank you very much**
